# Exploring the promise land of 7 T for CMR with T-PAT accelerated imaging techniques – first results for real time cardiac function and tagging in volunteers

**DOI:** 10.1186/1532-429X-11-S1-P146

**Published:** 2009-01-28

**Authors:** Gregory Metzger, Peter Weale, Lance DelaBarre, Patrick Bolan, Sven Zuehlsdorff, Sonia Nielles-Vallespin, Pierre-Francois Van de Moortele, Carl J Snyder, Edward J Auerbach, J Thomas Vaughan, Kamil Ugurbil, Renate Jerecic

**Affiliations:** 1 CMRR, Minneapolis, IL USA; 2 Siemens Medical Solutions, Chicago, IL USA; 3 Siemens Healthcare, Erlangen, Germany

**Keywords:** Temporal Resolution, Cine Imaging, High Acceleration Factor, Complete Cardiac Cycle, Volumetric Coverage

## Introduction

CMR faces many challenges at 7 T, including increased magnetic susceptibility, increased power deposition and non-uniform B1 distribution. Particularly, non-uniformities in B1 become a challenge beyond 3 Tesla, as the radio-frequency wavelength becomes smaller than the size of the imaged object.

On the other hand the wish for higher spatial and temporal resolution, volumetric coverage and short scan times, ideally all at once, can only be satisfied to some extent, even at 3 T, by using parallel imaging techniques until the SNR limit is reached. Therefore the increased SNR and prolonged T1 relaxation times of 7 T are very tempting as a way to further push the limits of cardiac function and tissue characterization in combination with parallel imaging, especially as it could be shown recently, that the technical and physical challenges mentioned above can be overcome and first cardiac images of healthy volunteers were obtained at 7 T [1].

## Purpose

The purpose of this study was to explore the feasibility of increased temporal and spatial resolution for cardiac function and tagging using k-t accelerated imaging techniques at 7 T in healthy volunteers

## Methods

### Hardware and adjustments

Three volunteers were imaged under a protocol approved by the University of Minnesota's IRB using a 7 T whole-body system (MAGNETOM 7 T, Siemens Healthcare, Germany) with the magnet provided by MAGNEX Scientific, UK. A home-built sixteen-channel, flexible, transceiver array coil was used, with 8 elements on the anterior and posterior part. Standard wireless VCG gating was used.

The B1-shimming procedure was performed, to optimize the RF-transmit efficiency over the heart. The transmit B1-field components of the independent coil elements were adjusted, to affect an approximate "constructive interference" of the short RF wavelengths over the region of the left ventricle as described in [2, 3]. SAR monitoring was done according to [4]

### Cardiac sequences

As a reference a typical clinical protocol was acquired for cardiac function and tagging using a segmented, breath hold, VCG triggered, retro-gated 2D cine FLASH sequence with a GRAPPA factor of 2, temporal resolution 40 ms, spatial resolution 2.3 mm inplane, slice thickness 3 mm for both sequences.

The tagging acquisition was then repeated using a T-PAT accelerated segmented 2D cine FLASH technique to maintain the spatial resolution and increase the temporal resolution to 19 ms using a T-GRAPPA factor of 3.

To show the potential of high spatio-temporal resolution cine imaging, real-time 2D cine FLASH images were acquired during free breathing, using T-PAT [5] with a T-GRAPPA factor 5, temporal resolution 68 ms and matched spatial resolution to the segmented acquisition.

All scans were acquired in short axis (sax) orientation. No surface coil normalization was used.

## Results

**Figure 1:**
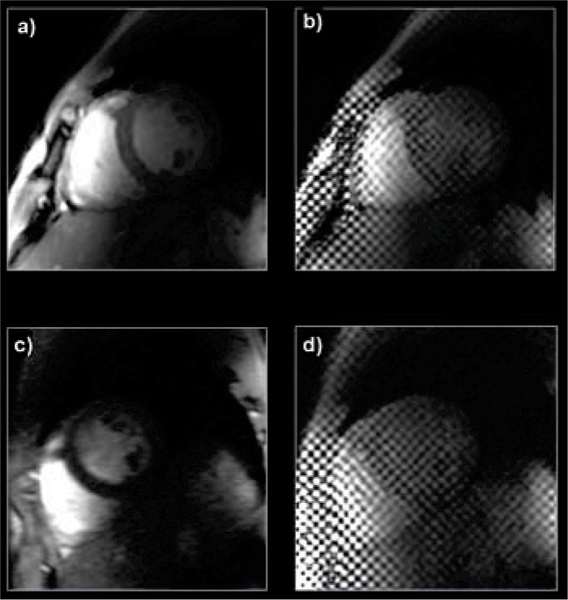
Figure 1

All scans could be completed successfully. A typical result is shown in Figure 1 a) and b) reference cine and tagging, c) rt cine, d) 19 ms tagging). The tag pattern remains persistent throughout the complete cardiac cycle, due to the increased T1. Image contrast between blood pool and myocardium is increased for FLASH compared to 1.5 T for the same reason. None of the sequences was limited by SAR using the sequence parameters described above.

## Conclusion

Real-time cine imaging and tagging with high temporal and spatial resolution is possible at 7 T with good image quality, without running into SAR limitations. This sets the basis for further protocol optimization going to even higher acceleration factors.

Further improvements in B1-shimming will allow a homogeneous excitation over a larger region-of-interest and therefore the extension of the protocol towards a whole heart coverage.
